# Platelet/high-density lipoprotein cholesterol ratio as a biomarker of depression in individuals with chronic opioid use

**DOI:** 10.3389/fpsyt.2025.1582449

**Published:** 2025-06-18

**Authors:** Qin Li, Chengxiang Liu, Shan Chen, Hong Chen

**Affiliations:** Second Hospital of Anhui Medical University, Hefei, China

**Keywords:** platelet/HDL cholesterol ratio, depression, chronic opioid use, biomarkers, inflammation, lipid metabolism, NHANES

## Abstract

**Background:**

The comorbidity of depression and opioid use is increasingly recognized as a significant public health concern. Chronic opioid use can alter biological systems, including lipid metabolism and inflammatory responses, potentially contributing to depressive symptoms. The platelet/high-density lipoprotein cholesterol ratio (PHR) has emerged as a biomarker associated with both cardiovascular and mental health outcomes. This study investigates the relationship between PHR and depression in individuals with chronic opioid use.

**Methods:**

A cross-sectional analysis was conducted using data from the National Health and Nutrition Examination Survey (NHANES) (2007–2018). A total of 843 participants with prescription opioid use were included. Depression was assessed using the Patient Health Questionnaire-9 (PHQ-9), with a score ≥10 indicating clinically significant depression. PHR was calculated from platelet counts and HDL cholesterol levels and categorized into quartiles. Weighted logistic regression and restricted cubic spline regression were employed to evaluate associations and potential nonlinear relationships, adjusting for demographic, socioeconomic, lifestyle, and clinical covariates.

**Results:**

Higher PHR quartiles were significantly associated with increased odds of depression, even after full adjustment for confounders (OR for Q3: 3.40; 95% CI: 1.95–5.94; OR for Q4: 4.12; 95% CI: 2.21–7.12). A nonlinear relationship was observed, with depression risk increasing sharply beyond a specific PHR threshold. Subgroup analyses revealed stronger associations in younger participants and those with obesity, with significant interaction effects for age and BMI.

**Conclusion:**

Elevated PHR is independently associated with depression in individuals with chronic opioid use, suggesting its potential as a biomarker for identifying at-risk populations. The findings underscore the need to address systemic inflammation and lipid dysregulation as part of integrated mental health care for opioid users.

## 1 Introduction

The association between opioid use and mental health disorders, particularly depression, has been a subject of extensive research in recent years (1). Opioids, especially prescription painkillers, are commonly used for the management of chronic pain but have been linked to numerous adverse outcomes, including the development of substance use disorders and mood disturbances (2). One of the key factors influencing the relationship between opioid use and depression is the alteration of various biological systems, including inflammation and lipid metabolism (3). Among these, the platelet/high-density lipoprotein cholesterol ratio (PHR) has emerged as a potential biomarker for cardiovascular and mental health disorders (4). This ratio is thought to reflect a state of systemic inflammation and dyslipidemia, both of which have been implicated in the pathogenesis of depression (5).

Opioid use, particularly chronic prescription opioid consumption, is known to have significant effects on various biological systems, including lipid metabolism and platelet activity. Recent studies have highlighted the bidirectional nature of this relationship, with opioid use exacerbating depressive symptoms and vice versa. Additionally, the potential role of biological markers in improving early identification and stratification of high-risk groups in depression, including treatment-resistant depression (TRD), has gained attention. Maina et al. (2023) emphasize the importance of biological markers in the management of TRD, which is highly relevant when considering the role of biomarkers like the platelet/high-density lipoprotein cholesterol ratio (PHR) in opioid users (6). Opioids can increase platelet aggregation, which is linked to inflammatory responses that may play a role in mood disturbances (7). Chronic opioid use, by altering lipid profiles and inducing inflammation, may lead to dysregulated platelet function and changes in the platelet/high-density lipoprotein cholesterol ratio (PHR), a marker associated with systemic inflammation and vascular health. In turn, these biological alterations could contribute to the development of depressive symptoms, as inflammation and lipid dysregulation have been implicated in the pathophysiology of depression (8). Opioid receptors, including the mu, delta, and kappa receptors, are expressed on the membranes of platelets. These receptors play a critical role in platelet aggregation and activation, which could contribute to the inflammatory processes associated with depression in individuals using opioids. In addition to inflammation, other neurotransmission systems, particularly the serotonergic system, are critical in the pathophysiology of depression. The serotonin transporter, present on both platelets and neurons, regulates serotonin uptake. Alterations in serotonin function, including reduced serotonin transporter activity, may impair platelet function and further contribute to depression by enhancing the inflammatory response and disrupting the balance of neurotransmitters. This relationship is particularly concerning in the context of opioid use, where these changes may be amplified. The PHR itself has been proposed as a marker of vascular dysfunction and inflammatory processes, which are common in individuals with both opioid use disorders and depression. Numerous studies have highlighted the bidirectional relationship between depression and inflammation, with both conditions exacerbating each other’s severity (9, 10). Similarly, emerging research has linked altered lipid profiles, including reduced HDL cholesterol levels and increased platelet aggregation, to an increased risk of depressive symptoms (11).

In the context of opioid use, it is plausible that chronic opioid consumption could lead to a dysregulation of lipid metabolism, contributing to changes in the PHR that may predispose individuals to depressive symptoms. This hypothesis is supported by evidence showing that individuals with opioid use disorders often exhibit altered lipid profiles and increased inflammatory markers (12). Furthermore, research suggests that depression itself can influence platelet activity and lipid metabolism, creating a complex interplay between these factors in individuals with opioid use history (13).

Recent epidemiological studies have underscored the importance of understanding how these biological markers interact with the neurobiological mechanisms of depression in opioid users. For example, a study by Macedo et al. found that individuals with a history of opioid misuse exhibited significantly higher platelet counts and lower HDL cholesterol levels, which were associated with more severe depressive symptoms (14). Additionally, the PHR has been shown to be a reliable marker of cardiovascular risk (15), which is often heightened in individuals with substance use disorders, particularly those using opioids.

Despite the growing interest in understanding the interplay between opioids, depression, and cardiovascular health, studies specifically examining the role of PHR in this context remain scarce. Research to date has primarily focused on PHR as a marker of cardiovascular risk, with limited investigations exploring its potential relevance to mental health, particularly in opioid users. While some evidence suggests that PHR might serve as an early indicator of depressive symptoms (16), its predictive value in the context of opioid-related disorders remains largely unexplored. Furthermore, the biological mechanisms linking PHR, opioid use, and depression are not well understood, highlighting the need for more targeted research to elucidate these complex interactions.

In this study, we aim to investigate the relationship between PHR and depression in a cohort of adults with a history of prescription opioid use between 2007 and 2018. We hypothesize that alterations in the PHR are associated with an increased risk of depression, due to underlying inflammation and lipid dysregulation induced by opioid use. Understanding this relationship could provide valuable insights into the complex biological interactions between opioids, depression, and cardiovascular risk, potentially leading to improved therapeutic strategies for individuals with opioid use disorders.

## 2 Methods

### 2.1 Study design and population

**Figure 1:**
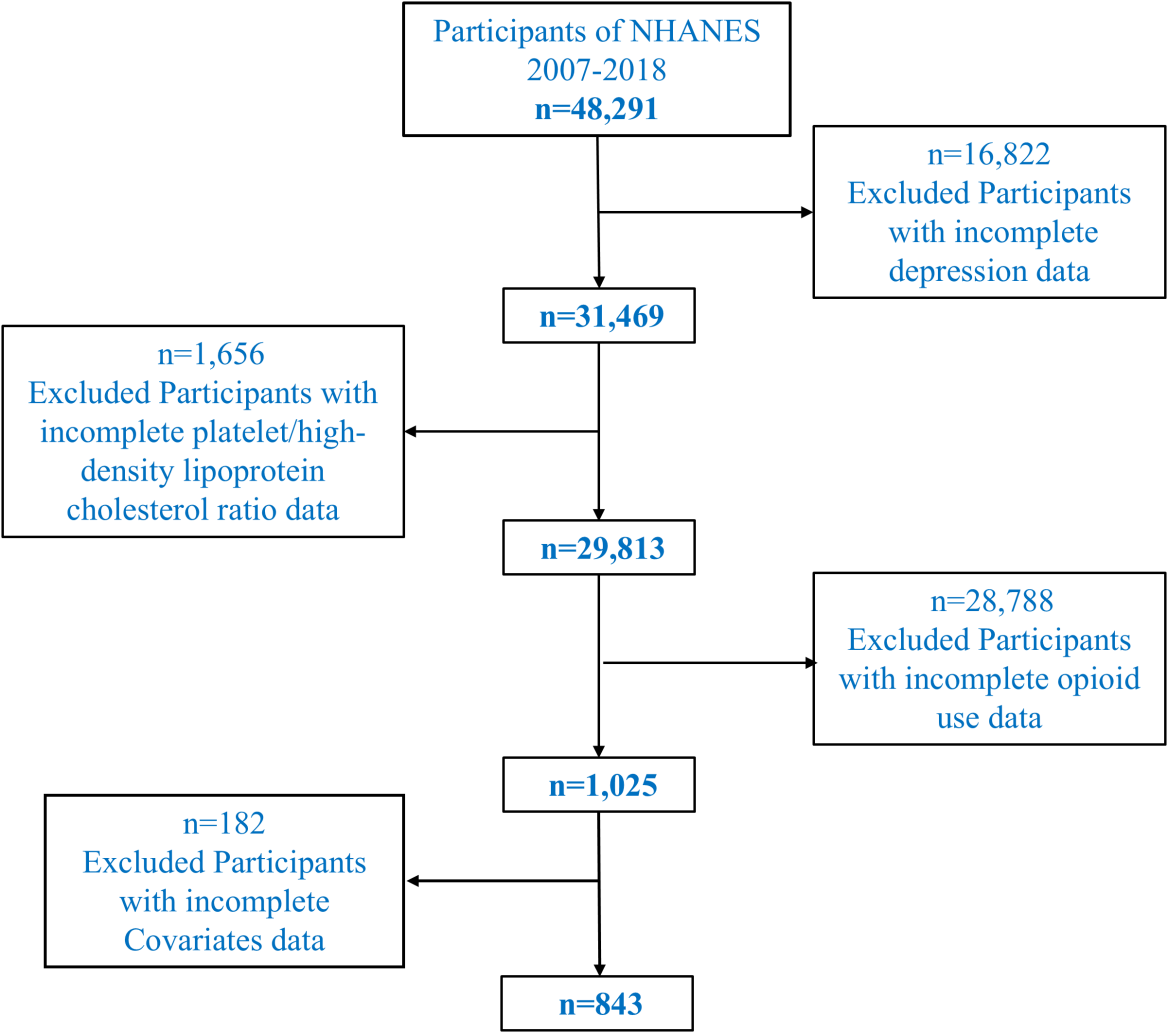
Population screening diagram.

This cross-sectional study utilized data from the National Health and Nutrition Examination Survey (NHANES) conducted between 2007 and 2018, a program designed to assess the health and nutritional status of the U.S. population through a complex, multistage probability sampling design. In the exclusion criteria, we specifically excluded participants who were undergoing pharmacological treatments that could influence depressive states or biochemical indicators, such as antidepressants, statins, and anti-inflammatory drugs. These medications can affect lipid profiles, platelet activity, and inflammatory markers, which are relevant to the study’s focus on platelet/high-density lipoprotein cholesterol ratio (PHR). To ensure the results accurately reflect the relationship between opioid use and depression, only individuals not currently on these treatments were included in the study. The initial dataset included 48,291 participants. To identify a relevant study population, we sequentially excluded individuals based on specific criteria. First, participants with missing data on depression, assessed using the Patient Health Questionnaire-9 (PHQ-9), were excluded (n = 16,822), leaving 31,469 participants. Second, individuals with incomplete laboratory data for calculating the platelet/high-density lipoprotein cholesterol ratio (PHR) were excluded (n = 1,656), resulting in 29,813 participants. Third, we identified 1,025 individuals who provided complete information on prescription opioid use. Chronic opioid use was defined as the regular use of prescription opioids for at least three months. Participants who reported using opioid medications such as oxycodone, hydrocodone, morphine, or fentanyl (whether oral, transdermal, or through other routes) were classified as chronic opioid users. The most common opioids in the study were oxycodone and hydrocodone, with oral consumption being the predominant route. Participants using opioid patches (e.g., fentanyl dermal patches) for therapeutic purposes or those using opioids recreationally (e.g., fentanyl or heroin) were also included in the chronic opioid use group. The type of opioid and the route of administration (oral, dermal, intravenous, or others) were recorded to allow for detailed analysis of the impact of opioid consumption on depression and biochemical markers. Lastly, participants with missing data for key covariates, including demographic, socioeconomic, lifestyle, and clinical variables, were excluded, yielding a final analytical sample of 843 participants. A flowchart illustrating the selection process is provided in **Figure 1**.

### 2.2 Depression assessment

Depressive symptoms were assessed using the Patient Health Questionnaire-9 (PHQ-9), a validated tool widely used in epidemiological studies. The PHQ-9 evaluates the frequency of nine depressive symptoms over the past two weeks, with response options ranging from 0 (“not at all”) to 3 (“nearly every day”). The total score ranges from 0 to 27, with higher scores indicating greater depressive symptomatology. For this study, a PHQ-9 score of 10 or higher was used to define clinically significant depression, consistent with its established sensitivity and specificity for diagnosing major depressive disorder (17). This binary classification allowed us to differentiate individuals with significant depressive symptoms from those without.

### 2.3 Platelet/high-density lipoprotein cholesterol ratio

The platelet/high-density lipoprotein cholesterol ratio (PHR) was calculated as the ratio of platelet count to HDL-C level, both of which were measured following NHANES standardized protocols. Platelet count, expressed as ×10^9^/L, was determined using a complete blood count (CBC) with a five-part differential analyzer, a reliable tool for hematological analysis. HDL-C concentration, expressed in mg/dL, was measured enzymatically after dextran sulfate-magnesium precipitation of other lipoproteins (18). The platelet/high-density lipoprotein cholesterol ratio (PHR) was calculated as the ratio of platelet count (×10^9^/L) to high-density lipoprotein cholesterol (HDL-C) level (mg/dL), both measured following NHANES standardized protocols. Specifically, the PHR is calculated using the following formula:


$$\text{PHR}=\text{Platelet count}(\times 10^{9}/\text{L})/\text{HDL}-\text{C}(\text{mg}/\text{dL})$$

All laboratory procedures adhered to NHANES quality control standards to ensure accuracy and reliability of the measurements. PHR was then derived as a continuous variable and subsequently categorized into quartiles based on the weighted distribution within the study population.

### 2.4 Covariates

Several covariates were included to account for potential confounders in the association between PHR and depression. Demographic variables comprised age (measured in years), gender (male or female), race/ethnicity (Mexican American, other Hispanic, non-Hispanic Black, non-Hispanic White, and other races), and marital status (married, widowed, divorced, separated, never married, or living with a partner). Socioeconomic factors included educational attainment (less than 9th grade, 9–11th grade, high school graduate, some college or associate degree, and college graduate or above) and poverty-income ratio (PIR, categorized as ≤1, 1–3, and >3). Lifestyle factors such as smoking status (current smoker or non-smoker) and alcohol consumption (yes or no, based on self-reported alcohol use in the past 12 months) were also considered. Clinical characteristics included hypertension, hyperlipidemia, diabetes (classified as yes, no, or borderline), body mass index (BMI; categorized as underweight, normal weight, overweight, and obese), and self-reported average sleep duration in hours. These covariates were selected based on their theoretical or empirical associations with both PHR and depression.

### 2.5 Statistical analysis

All statistical analyses were conducted using SPSS version 27.0 (IBM Corp., Armonk, NY) and R version 4.4.2, with the application of NHANES survey weights to account for the complex sampling design and ensure nationally representative estimates. Continuous variables were expressed as means with standard deviations (SDs), while categorical variables were summarized as frequencies and percentages. Differences between individuals with and without depression were assessed using the independent-samples t-test for continuous variables and the chi-square test for categorical variables. To evaluate the association between PHR and depression, weighted logistic regression models were constructed with three levels of adjustment. Model 1 adjusted for age and gender. Model 2 included additional adjustments for race/ethnicity, marital status, education level, and PIR. Model 3 further adjusted for smoking, alcohol consumption, hypertension, hyperlipidemia, diabetes, BMI, and sleep duration. Results were expressed as odds ratios (ORs) with 95% confidence intervals (CIs), with the lowest PHR quartile (Q1) serving as the reference group.

Restricted cubic spline regression was employed to explore potential nonlinear relationships between PHR and depression. Knots were placed at the 10th, 50th, and 90th percentiles of PHR to provide a flexible yet parsimonious model for capturing dose-response relationships. Subgroup analyses were performed to investigate whether the association between PHR and depression varied by key factors such as gender, age, and BMI. Interaction terms were tested in the logistic regression models to formally evaluate effect modification, and results from these analyses were visualized using forest plots.

### 2.6 Ethical considerations

The NHANES study was approved by the National Center for Health Statistics (NCHS) Research Ethics Review Board. All participants provided written informed consent prior to participation. The present study, being a secondary analysis of publicly available and de-identified data, was exempt from additional ethical review by our institutional ethics committee.

## 3 Results

### 3.1 Baseline characteristics of the study participants

The baseline characteristics of the 843 participants included in the final analysis are presented in **Table 1**. The table is divided into two sections: sociodemographic variables and clinical variables. Sociodemographic variables include age, gender, race/ethnicity, and marital status, while clinical variables are further categorized into non-pathological personal history (e.g., smoking status, alcohol consumption) and pathological history (e.g., hypertension, hyperlipidemia, diabetes). Comorbidities are classified under pathological history to differentiate them from non-pathological factors. The mean age of the participants was 56.72 ± 14.93 years, with individuals experiencing depression being significantly younger than those without depression (52.25 ± 14.49 *vs*. 58.54 ± 13.73 years, p = 0.002). A quartile graph by gender and for the total population was used to visualize the distribution of participants’ ages. This distribution was further examined to assess the potential impact of menopause and climacteric symptoms in women, which are strongly associated with depression. Among the study population, 47.9% were female, and while gender distribution did not differ significantly between groups with and without depression (p = 0.149), a higher proportion of depressed individuals were female (13.9%) compared to males (15.1%). Race and ethnicity also showed marginal differences (p = 0.057), with a larger percentage of non-Hispanic Black individuals (54.7%) comprising the total sample, and depressed individuals being more represented in this group (16.7%).

**Table 1:** Baseline characteristics of the study participants.

Characteristics	Overall	Depression		*P*-value
		No	Yes	
n	843	599	244	
**Age, years**	56.72±14.93	58.54±13.73	52.25±14.49	0.002
**Gender, n (%)**				0.149
Female	404(47.9%)	287(34.0%)	117(13.9%)	
Male	439(52.1%)	312(37.0%)	127(15.1%)	
**Race, n (%)**				0.057
Mexican American	86(10.2%)	55(6.5%)	31(3.7%)	
Other Hispanic	71(8.4%)	48(5.7%)	23(2.7%)	
Non-Hispanic Black	461(54.7%)	320(38.0%)	141(16.7%)	
Non-Hispanic White	169(20.0%)	137(16.3%)	32(3.8%)	
Other races	56(6.6%)	39(4.6%)	17(2.0%)	
**Education, n (%)**				0.542
Less than 9th grade	83(9.8%)	57(6.8%)	26(3.1%)	
9-11th grade	150(17.8%)	103(12.2%)	47(5.6%)	
High school graduate	211(25.0%)	142(16.8%)	69(8.2%)	
Some college or AA degree	280(33.2%)	199(23.6%)	81(9.6%)	
College graduate or above	119(14.1%)	98(11.6%)	21(2.5%)	
**Marital Status, n(%)**				0.815
Married	415(49.2%)	297(35.2%)	118(14.0%)	
Widowed	76(9.0%)	63(7.5%)	13(1.5%)	
Divorced	151(17.9%)	108(12.8%)	43(5.1%)	
Separated	48(5.7%)	31(3.7%)	17(2.0%)	
Never married	105(12.5%)	68(8.1%)	37(4.4%)	
Living with partner	48(5.7%)	32(3.8%)	16(1.9%)	
**PIR, n (%)**				0.209
<=1	247(29.3%)	160(19.0%)	87(10.3%)	
1-3	376(44.6%)	264(31.3%)	112(13.3%)	
>3	220(26.1%)	175(20.8%)	45(5.3%)	
**Smoke, n (%)**				0.007
Yes	557(66.1%)	374(44.4%)	183(21.7%)	
No	286(33.9%)	225(26.7%)	61(7.2%)	
**Alcohol Use, n (%)**				0.079
Yes	644(76.4%)	463(54.9%)	181(21.5%)	
No	199(23.6%)	136(16.1%)	63(7.5%)	
**Hypertension, n (%)**				0.015
Yes	509(60.4%)	359(42.6%)	150(17.8%)	
No	334(39.6%)	240(28.5%)	94(11.2%)	
**Hyperlipidemia, n (%)**				0.046
Yes	438(52.0%)	297(35.2%)	141(16.7%)	
No	405(48.0%)	302(35.8%)	103(12.2%)	
**Diabetes, n (%)**				<0.001
Yes	209(24.8%)	144(17.1%)	65(7.7%)	
Borderline	18(2.1%)	16(1.9%)	2(0.2%)	
No	615(73.0%)	438(52.0%)	177(21.0%)	
**BMI, n (%)**				<0.001
Underweight	15(1.8%)	10(1.2%)	5(0.6%)	
Normal weight	181(21.5%)	151(17.9%)	30(3.6%)	
Overweight	246(29.2%)	179(21.2%)	67(7.9%)	
Obesity	401(47.6%)	259(30.7%)	142(16.8%)	

Educational level and marital status did not show significant differences between groups (p = 0.542 and p = 0.815, respectively). This simplification allowed for clearer assessment of whether companionship influences depression. However, lifestyle and clinical characteristics highlighted notable disparities. Smokers were significantly more likely to be depressed compared to non-smokers (21.7% *vs*. 7.2%, p = 0.007). Similarly, hypertension (p = 0.015), hyperlipidemia (p = 0.046), and diabetes (p < 0.001) were more prevalent in depressed individuals. Body mass index (BMI) distributions also differed significantly (p < 0.001), with obesity being more common among depressed individuals (16.8%) compared to those without depression.

### 3.2 Association between PHR and depression in individuals with chronic opioid use

Weighted logistic regression analyses assessing the association between PHR quartiles and depression are summarized in **Table 2**. In the unadjusted model, higher PHR quartiles were positively associated with depression. After adjusting for age and gender (Model 1), participants in the third quartile (Q3) had significantly higher odds of depression compared to the lowest quartile (OR: 3.83, 95% CI: 2.23–5.55, p < 0.001). This association remained robust in Model 2, which additionally adjusted for race, marital status, education, and PIR (OR: 3.62, 95% CI: 2.09–6.24, p < 0.001), and in Model 3, which further controlled for lifestyle and clinical variables (OR: 3.40, 95% CI: 1.95–5.94, p < 0.001). Participants in the highest quartile (Q4) also exhibited significantly higher odds of depression compared to Q1 across all models, with a consistent trend (p for trend < 0.001). These findings suggest a strong and independent association between elevated PHR and depression among individuals with opioid use.

**Table 2:** Weighted logistic regression analyses of association between the platelet/high-density lipoprotein cholesterol ratio and depression in Opioid-Using Populations.

PHR	Model 1		Model 2		Model 3	
	OR 95%CI	P value	OR 95%CI	P value	OR 95%CI	P value
Q1	Ref		Ref		Ref	
Q2	0.55(0.27,1.16)	0.118	0.52(0.25,1.09)	0.083	0.50(0.24,1.06)	0.071
Q3	3.83(2.23,5.55)	<0.001	3.6192.09,6.24)	<0.001	3.40(1.95,5.94)	<0.001
Q4	2.09(1.22,3.58)	<0.001	1.97(1.13,3.42)	<0.001	1.81(1.03,3.19)	<0.001
p for trend		<0.001		<0.001		<0.001

Model 1: Adjusted for gender, age.

Model 2: Additionally, adjusted for race, marital status, education and poverty-income ratio.

Model 3: Additionally, adjusted for smoke, alcohol Use, hypertension, hyperlipidemia, diabetes, BMI, sleep time.

### 3.3 Nonlinear relationship between PHR and depression

**Figure 2:**
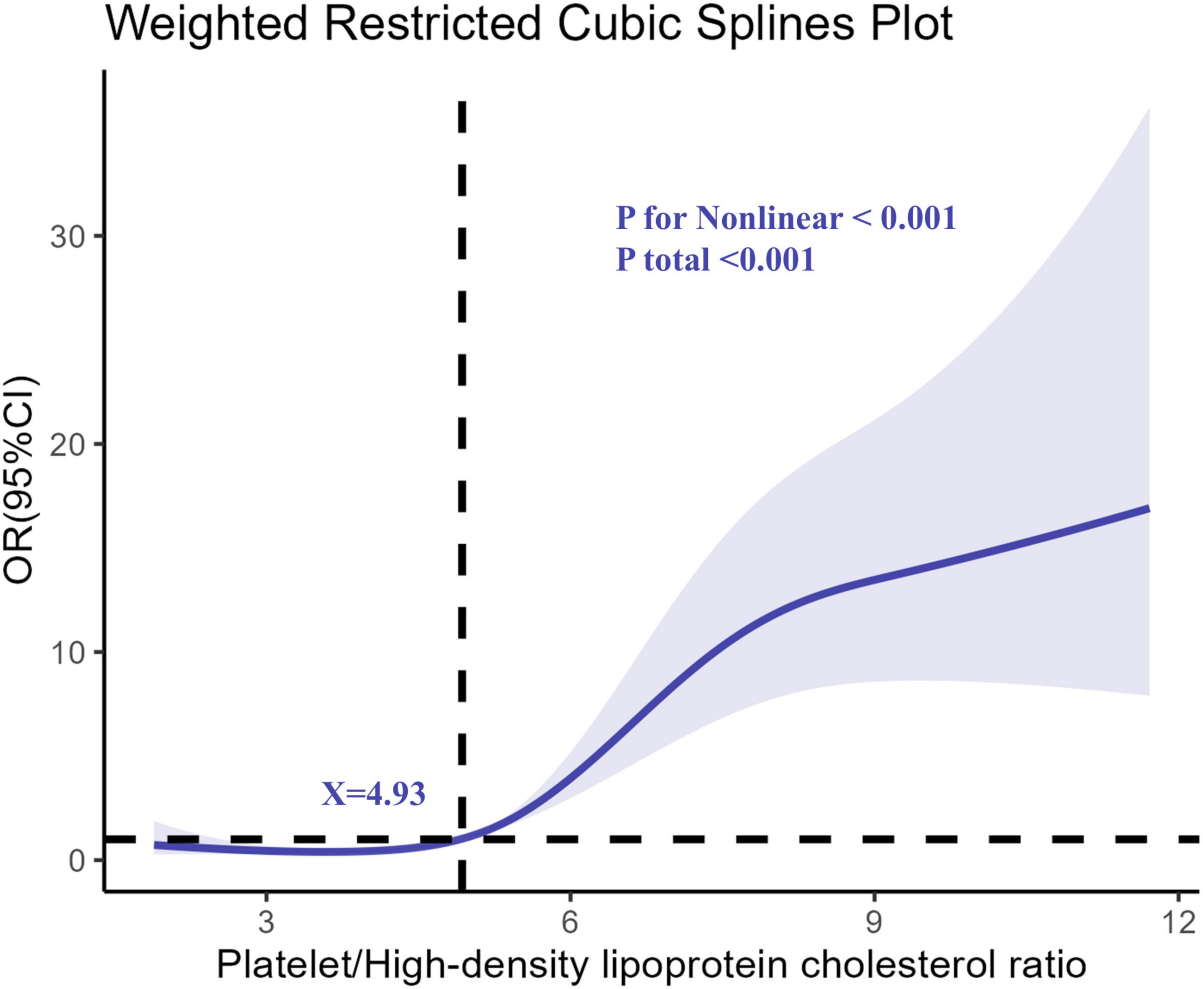
The nonlinear relationship between PHR and the odds of depression.

The restricted cubic spline analysis revealed a nonlinear relationship between PHR and the odds of depression, as shown in **Figure 2**. The association became particularly pronounced at higher levels of PHR, indicating a dose-response relationship. The risk of depression remained relatively stable at lower PHR levels but increased sharply after a specific threshold, suggesting that elevated PHR might serve as a critical biomarker for identifying individuals at greater risk of depression.

### 3.4 Subgroup and interaction analyses

**Figure 3:**
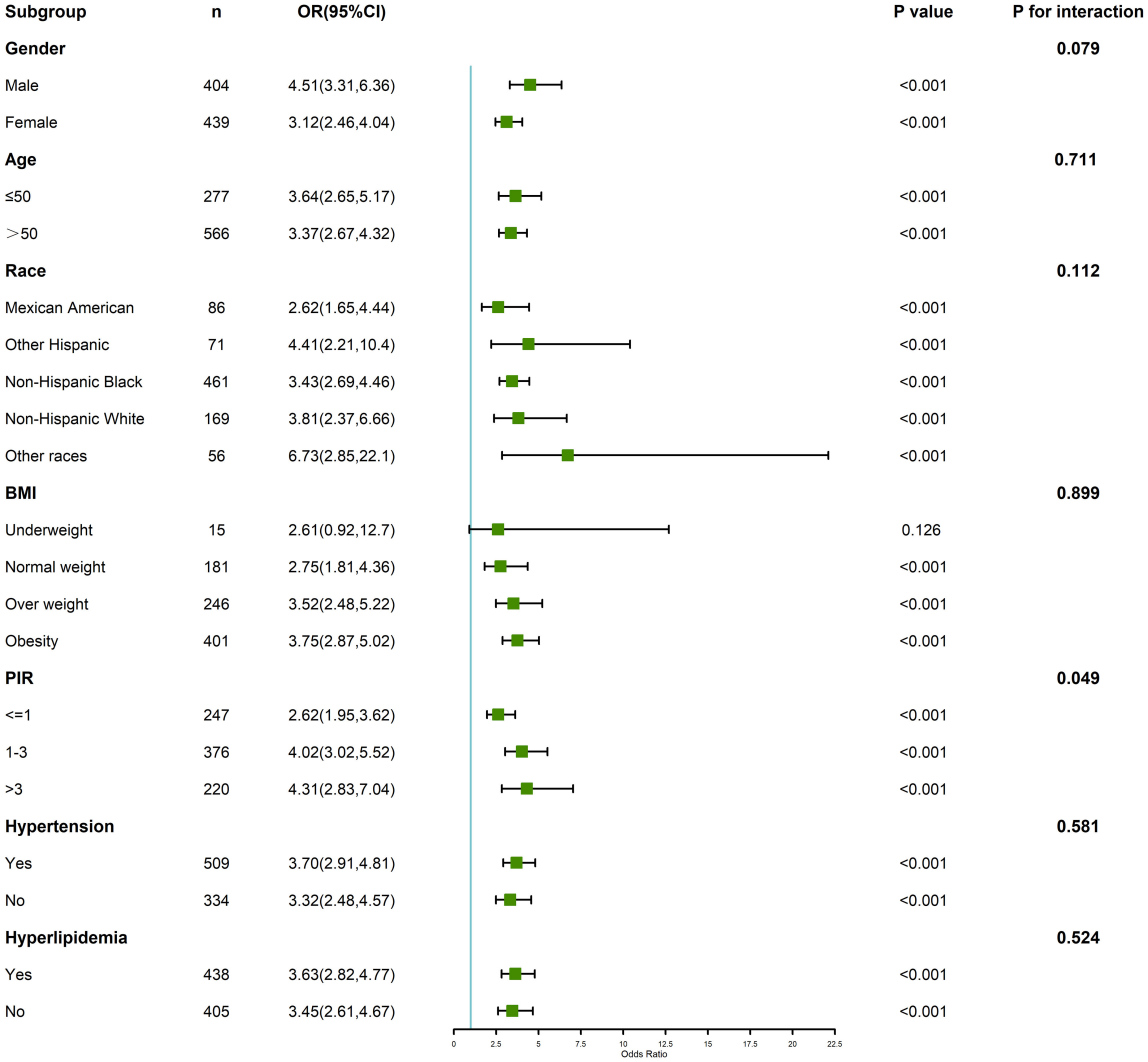
Forest plot of subgroup analysis and interaction analysis.

Subgroup analyses stratified by age, gender, BMI, and other key covariates are summarized in **Figure 3**. The association between PHR and depression was consistent across most subgroups but exhibited variations in magnitude. For instance, the effect of higher PHR on depression was more pronounced in younger individuals (age < 60 years) and in those with obesity (BMI ≥ 30). The interaction analyses revealed significant effect modification by age (p for interaction = 0.01) and BMI (p for interaction = 0.03), indicating that these factors may amplify the impact of elevated PHR on depression risk. Gender-stratified analysis showed a slightly stronger association among females compared to males, although this interaction did not reach statistical significance (p for interaction = 0.08). These results highlight the potential importance of individual-level characteristics in modifying the relationship between PHR and depression.

## 4 Discussion

Depression, a common comorbidity among opioid users, is often mediated by biological alterations in inflammatory and metabolic pathways (19, 20). Platelet/high-density lipoprotein cholesterol ratio (PHR), a novel biomarker that integrates inflammatory and lipid profiles, offers a unique lens to understand these mechanisms. In this study, PHR was examined as a potential biomarker for depression among opioid users, contributing to a deeper understanding of how systemic inflammation and lipid metabolism may influence mental health outcomes.

A plausible mechanism underlying the association between PHR and depression lies in the role of chronic inflammation. Elevated platelet counts and reduced HDL cholesterol levels, which define higher PHR values, are both hallmarks of systemic inflammation. One possible mechanism underlying the increase in platelet counts is the activation of opioid receptors on platelet membranes. Opioids, particularly those commonly prescribed for pain management, may bind to these receptors and stimulate platelet aggregation. This process could lead to increased platelet counts and contribute to the inflammatory state observed in opioid users (21, 22). Chronic inflammation is well established as a key factor in the development of depression, and it can disrupt neurotransmitter pathways, alter hypothalamic-pituitary-adrenal (HPA) axis function, and impair neurogenesis. However, the mechanisms by which opioid use induces neuroinflammation are not fully understood. It is important to distinguish between peripheral and central nervous system mechanisms. While peripheral inflammation has been widely studied, opioids may also activate neuroinflammatory pathways in the brain. Previous research has shown that opioids can increase the production of pro-inflammatory cytokines, such as prostaglandins, in the central nervous system, which may contribute to mood disturbances and depression (23, 24). HDL cholesterol, beyond its lipid-transport role, has anti-inflammatory properties and modulates endothelial function, oxidative stress, and immune responses (25, 26). Thus, reduced HDL levels in individuals with high PHR could exacerbate inflammatory damage, creating a feedback loop that perpetuates both cardiovascular and neuropsychiatric risks.

Opioid use further complicates this interplay. Chronic opioid consumption induces immune dysregulation, increasing pro-inflammatory cytokines such as interleukin-6 (IL-6) and tumor necrosis factor-alpha (TNF-α), while simultaneously suppressing anti-inflammatory mechanisms (27–29). Opioid-induced reductions in HDL levels have also been documented, likely driven by alterations in hepatic lipid metabolism and peripheral immune activity (30, 31). These dual effects may heighten the vulnerability of opioid users to both cardiovascular and mental health disorders. The observed relationship between PHR and depression may therefore reflect a broader, multifaceted disruption of systemic homeostasis caused by opioid use.

From a comparative perspective, prior research has identified PHR as a marker of cardiovascular disease risk (32), with growing evidence linking it to psychiatric outcomes. While studies have highlighted the role of inflammatory markers such as CRP and IL-6 in depression, PHR offers distinct advantages by integrating two measurable and routinely assessed parameters: platelet count and HDL cholesterol (33–35). Unlike standalone markers, PHR captures a composite picture of systemic inflammation and lipid dysregulation, making it particularly relevant in populations with overlapping risks, such as opioid users. This study extends the application of PHR into a novel context, emphasizing its potential utility in mental health research and clinical practice.

Despite these strengths, the relationship between inflammation, lipid metabolism, and depression is complex and not fully understood. For instance, The nonlinear association observed in this study suggests that PHR influences depression risk only above a certain threshold, which may reflect the point at which the inflammatory response becomes significant enough to affect mood regulation. This threshold may be influenced by factors such as resilience and allostatic overload, which are common in stress-related depressive disorders. It is crucial to recognize that opioid treatment serves as an additional factor contributing to depression development, and individuals with high PHR may represent those at risk for allostatic overload, where the body’s ability to adapt to chronic stressors is compromised. The PHR index could therefore be useful in identifying individuals who may be more vulnerable to depression due to both systemic inflammation and the stress imposed by opioid use (36, 37). These pathways may interact with inflammation and lipid metabolism to influence mental health outcomes (38). Future research should integrate a broader range of biomarkers to disentangle these overlapping mechanisms.

An important aspect to consider in understanding the heterogeneity of depression risk among opioid users is the role of temperament traits. Recent studies have highlighted the significance of affective temperaments in modulating individual susceptibility to depressive symptoms. For example, Favaretto et al. (2024) (39) provide a comprehensive synthesis of clinical experiences and empirical evidence, emphasizing how distinct affective temperaments can either mitigate or exacerbate the psychological impact of chronic stressors, including opioid use. This perspective aligns with our findings, as individuals with certain temperament profiles may experience a heightened inflammatory response or altered lipid metabolism, thereby increasing their vulnerability to depression. Understanding these temperament-linked differences could inform personalized prevention and intervention strategies in clinical practice.

In addition to biological mechanisms, it is critical to consider the clinical and public health implications of these findings. Depression among opioid users is often underdiagnosed, due in part to overlapping symptoms with opioid withdrawal and the stigma surrounding both conditions (40, 41). Identifying simple, cost-effective biomarkers such as PHR could facilitate earlier recognition and intervention, particularly in resource-limited settings. Moreover, the association between PHR and depression underscores the importance of addressing systemic inflammation and lipid dysregulation as part of comprehensive mental health care strategies. Anti-inflammatory therapies, HDL-raising interventions, and lifestyle modifications targeting these pathways may hold promise for mitigating depression risk in this population (42, 43).

Nevertheless, this study is not without limitations. The cross-sectional design precludes causal inference, leaving open the question of whether elevated PHR is a cause or consequence of depression. Additionally, the broad age range of the studied population, along with factors such as menopause and climacteric states in women, may act as confounding variables influencing the results. Life stressors and comorbid conditions such as diabetes, which were not fully accounted for in this analysis, may also contribute to depression and alter PHR levels. Future studies should aim to address these factors more comprehensively. Residual confounding by unmeasured variables, such as dietary patterns, physical activity, and genetic predispositions, cannot be ruled out (44). Additionally, while PHR integrates key markers of inflammation and lipid metabolism, it does not capture the full spectrum of biological processes involved in depression. Longitudinal studies are needed to validate these findings and to explore the potential of PHR as a predictive marker for depression onset or treatment response (45).

## 5 Conclusion

In conclusion, this study provides valuable insights into the relationship between systemic inflammation, lipid dysregulation, and depression in opioid users, suggesting that PHR could serve as a potential biomarker for identifying individuals at risk for depression. However, further research, including longitudinal studies and clinical trials, is needed to validate the use of PHR as a predictive marker for depression and to better understand the underlying mechanisms. By integrating insights from cardiovascular and psychiatric research, it offers a novel framework for understanding and addressing the unique challenges faced by individuals with opioid use. Further investigation is warranted to elucidate the mechanisms underlying these associations and to translate these findings into targeted interventions that improve both mental and physical health outcomes in this vulnerable population.

## Data Availability

The raw data supporting the conclusions of this article will be made available by the authors, without undue reservation.
